# Meta-Analysis of Chinese Traditional Medicine Bushen Huoxue Prescription for Endometriosis Treatment

**DOI:** 10.1155/2017/5416423

**Published:** 2017-11-23

**Authors:** Jing Shan, Wen Cheng, Dong-xia Zhai, Dan-ying Zhang, Rui-pin Yao, Ling-ling Bai, Zai-long Cai, Yu-huan Liu, Chao-qin Yu

**Affiliations:** ^1^Shanghai University of Traditional Chinese Medicine, Shanghai, China; ^2^Department of Gynecology of Traditional Chinese Medicine, Changhai Hospital, Second Military Medical University, Shanghai, China; ^3^Department of Biochemistry and Molecular Biology, Second Military Medical University, Shanghai, China; ^4^Department of Gynecology and Obstetrics, Changhai Hospital, Second Military Medical University, Shanghai, China

## Abstract

**Objectives:**

To evaluate the efficacy and safety of Bushen Huoxue prescription (BSHXP) for endometriosis.

**Methods:**

A meta-analysis was performed, and studies were searched from the seven databases from the date of database establishment to April 30, 2017. Randomized controlled trials (RCTs) that explored the efficacy and safety of BSHXP for patients with endometriosis were included. Two assessors independently reviewed each trial. The Cochrane Risk of Bias assessment tool was used for quality assessment.

**Results:**

In the 13 included studies, the total effectiveness rates of BSHXP were higher than those of Western medicine (RR, 1.55; 95% CI, 1.03–2.32; *P* = 0.04), but the dysmenorrhea alleviation rates of the two treatments did not significantly differ (RR, 1.28; 95% CI, 0.70–2.34; *P* = 0.42). The pregnancy rates of BSHXP were also higher than those of hormone therapy (RR, 1.99; 95% CI, 1.17–3.39; *P* = 0.01). However, whether BSHXP is more effective than Western medicine in diminishing endometriotic cyst remains unknown.

**Conclusions:**

Our study provides evidence that BSHXP is effective and safe for endometriosis, but this evidence is inconclusive because of the low methodological quality of the included RCTs. Our findings suggest that BSHXP is an alternative drug for endometriosis, but it should be further examined in future clinical research.

## 1. Introduction

Endometriosis is a gynecological disease characterized by the presence and growth of ectopic endometrial tissue and often associated with inflammation, chronic pain, and infertility [1]. Compared with normal females, affected women are at a twofold to threefold increased risk of developing ovarian cancer, although endometriosis is a seemingly benign disease [2]. The prevalence of endometriosis approximately ranges from 2% to 10% of reproductive aged women and to 50% of infertile women [3, 4]. Endometriosis can significantly influence the quality of life, ability to work, and fertility of reproductive aged women [5]. However, endometriosis is poorly understood, and the incidence of endometriosis likely increases [6]. The definitive treatment for endometriosis remains a difficult problem [7]. Current treatment strategies aim to diminish lesions, reduce pain, improve reproductive capacity, and reduce and avoid the chance of recurrence [8]. Various treatment methods, including medical, surgical, and alternative approaches, have been administered to patients with endometriosis [9]. Medications used in clinical settings commonly include hormonal contraceptives, GnRH agonists, aromatase inhibitors, nonsteroidal anti-inflammatory drugs, and progestins [10]. These medications can alleviate the symptoms of endometriosis, but these benefits disappear upon drug discontinuation [11]. Most of these options yield some side effects, such as insomnia, weight gain, rash, mood swings, depression, nausea, headache, bone density loss, and venous thrombosis [12]. Considering that endometriosis is a chronic condition, researchers should develop medical therapy that can be used continuously for symptom control [13]. Alternative therapies for endometriosis have also been developed. Among various therapies for endometriosis, traditional Chinese medicine (TCM) has been preferred by women with endometriosis [14].

According to TCM theory, kidney deficiency and blood stasis are a common syndrome of endometriosis, which is usually characterized by various conditions, such as dysmenorrhea, back pain, blood clots in menstruation, cold hands and feet, and fatigue [15]. Endometriosis is effectively treated with Bushen Huoxue prescription (BSHXP). In vivo tests have shown that BSHXP can inhibit the invasion of endometrial stromal cells and the expression of MMP-9 [16]. Animal experiments have demonstrated that BSHXP can promote the ability of apoptotic cells in endometriosis to hasten the atrophy and regression of endometriotic tissues [17].

However, data supporting the validity of this treatment are insufficient. This systematic review aimed to evaluate the efficacy and safety of BSHXP as a treatment for endometriosis by integrating different outcomes from clinical studies.

## 2. Methods and Materials

### 2.1. Methods

This systematic review was performed according to the Preferred Reporting Items for Systematic Review and Meta-Analyses Statement [18].

### 2.2. Study Selection

#### 2.2.1. Types of Studies

All of the RCTs reporting the application of BSHXP for the treatment of endometriosis were included. No limitations on publication status or language were set. Non-RCTs or animal studies were excluded.

#### 2.2.2. Types of Participants

Only female patients who satisfied at least one of the current diagnostic standards of endometriosis and kidney deficiency and blood stasis syndrome were included. No limitations on ethnicity or nationality were set. Patients with adenomyosis were excluded.

#### 2.2.3. Types of Interventions

RCTs that observed and compared the effects of BSHXP and Western medicine were identified. Patients in the treatment group were given a kidney-tonifying and blood-activating formula. Patients in the control group were treated with Western medicine. Patients were excluded when the trials included other cointerventions, including another herbal formula. We did not set limitations on dosages and routes of the administration of the traditional Chinese herbs.

#### 2.2.4. Types of Outcome Measures

The primary outcomes in this study were the total effectiveness rate, dysmenorrhea alleviation rate, and pregnancy rate. The secondary outcomes were changes in the size of endometriotic cyst and adverse events. The total effectiveness rate was assessed according to the evaluation criteria of the Guidelines of Clinical Research of New Drugs of Traditional Chinese Medicine (Table 1).

### 2.3. Search Strategy

Electronic searches were conducted in the Cochrane Central Register of Controlled Trials (CENTRAL, 1996–April 2017), EMBASE (1966–April 2017), PubMed (1959–April 2017), the Chinese National Knowledge Infrastructure (CNKI, 1979–April 2017), the Wanfang Database (1959–April 2017), the Chinese Scientific Journal Database (VIP, 1989–April 2017), and the Chinese Biomedical Literature Database (CBM, 1978–April 2017) from the date of database establishment to April 30, 2017, by two reviewers (J. Shan and W. Cheng). The references of the identified studies and ongoing registered clinical trials were also manually searched to retrieve unpublished articles. No restriction on language or publication status was set. The search terms for the literature search were as follows: (“endometriosis” OR “dysmenorrhea” OR “dyspareunia” OR “pelvic pain”) AND (“tonifying kidney and activating blood” OR “bu shen” OR “wen shen” OR “zi shen” OR “huo xue” OR “qu yu” OR “xiao zheng”) AND (“clinical trial” OR “randomized controlled trial” OR “randomised controlled trial”).

### 2.4. Data Extraction

Two reviewers (Dong-xia Zhai and Dan-ying Zhang) extracted basic information independently by using a standardized data extraction form. Disagreements were resolved by discussion or consensus with a third reviewer (Chao-qin Yu). Some important data, including first author's name, publication year, sample size, treatment interventions and control groups, BSHXP composition, treatment duration, outcome measures, and adverse effects, were extracted from primary trials. If a study was incomprehensive or uncertain, we then contacted the corresponding author by telephone, email, and fax to obtain the correct data.

### 2.5. Assessment of Risk of Bias

The risk of bias of the eligible trials was assessed by two independent authors using the Cochrane Risk of Bias tool [19]. Disagreements were resolved by obtaining consensus with a third reviewer (Chao-qin Yu). The risk of bias was evaluated on the basis of these domains: (1) selection bias (adequate sequence generation and concealment of allocation); (2) performance bias (blinding of the investigator and blinding of the assessor); (3) detection bias (blinding of the assessor); (4) attrition bias (incomplete outcome data addressed); (5) reporting bias (free of selective reporting); and (6) other sources of bias (other potential threats to validity).

### 2.6. Data Analyses

BSHXP and Western medicine were compared in this review. Outcome measures after treatment were presented as risk ratio (RR) with 95% CI for dichotomous outcomes. Cochrane's *P* values and *I*^2^ tests were determined to examine the level of heterogeneity between trials. A random-effects model was used to evaluate the effects of BSHXP on endometriosis if *I*^2^ > 50% or *P* < 0.1. Otherwise, a fixed-effects model was utilized. *P* < 0.05 was considered statistically significant. Data were subjected to meta-analysis by using Review Manager 5.3 (Cochrane Community, London, United Kingdom, 2014).

## 3. Results

### 3.1. Study Selection

**Figure 1:**
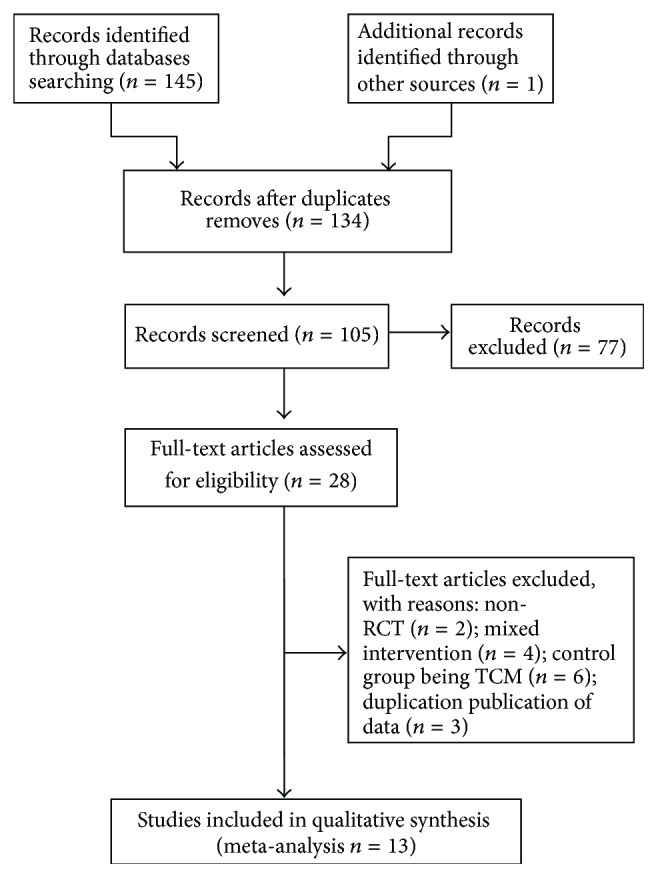
Flow diagram of study selection and identification.

A total of 146 studies were collected through document retrieval. Of these studies, 12 duplicate publications were removed. After their titles and abstracts were scanned, 77 articles were also excluded because they were clinical experience reports, case studies, and animal research. The 28 remaining full-text articles were further analyzed, but 15 trials were also eliminated because of the following: 2 non-RCT studies, 3 articles with duplicate publication of data, 4 articles with mixed interventions, and 6 articles with a control group containing herbal therapies. Finally, 13 eligible studies involving 936 patients with endometriosis were included in our meta-analysis (Figure 1).

### 3.2. Study Characteristics

The basic information of the included RCTs is summarized in Table 2. This meta-analysis included 13 trials. Of these trials, 12 were published in Chinese and 1 was presented in English. Of the 936 patients with endometriosis enrolled in these trials, 492 were assigned to the treatment group and 444 were designated in the control group. The sample sizes of these trials ranged from 48 to 120. The baseline in all of the trials did not significantly differ.

All of the trials involved two-arm designs: treatment group versus control group. Patients in the treatment group were treated with BSHXP (Table 3), while patients in the control group were administered with Western medicine, including mifepristone, Diphereline, gestrinone, and danazol. Treatment duration was 3 or 6 months. Of the 13 trials, 3 indicated dropouts [23, 29, 30]; 10 reported the total effective rates [21–30]; 7 presented the dysmenorrhea alleviation rates [24, 25, 27, 29–32]; 3 described the pregnancy rates [20, 31, 32]; and 6 discussed adverse effects [20, 21, 24, 29, 30, 32].

### 3.3. Risk of Bias Assessment

**Figure 2:**
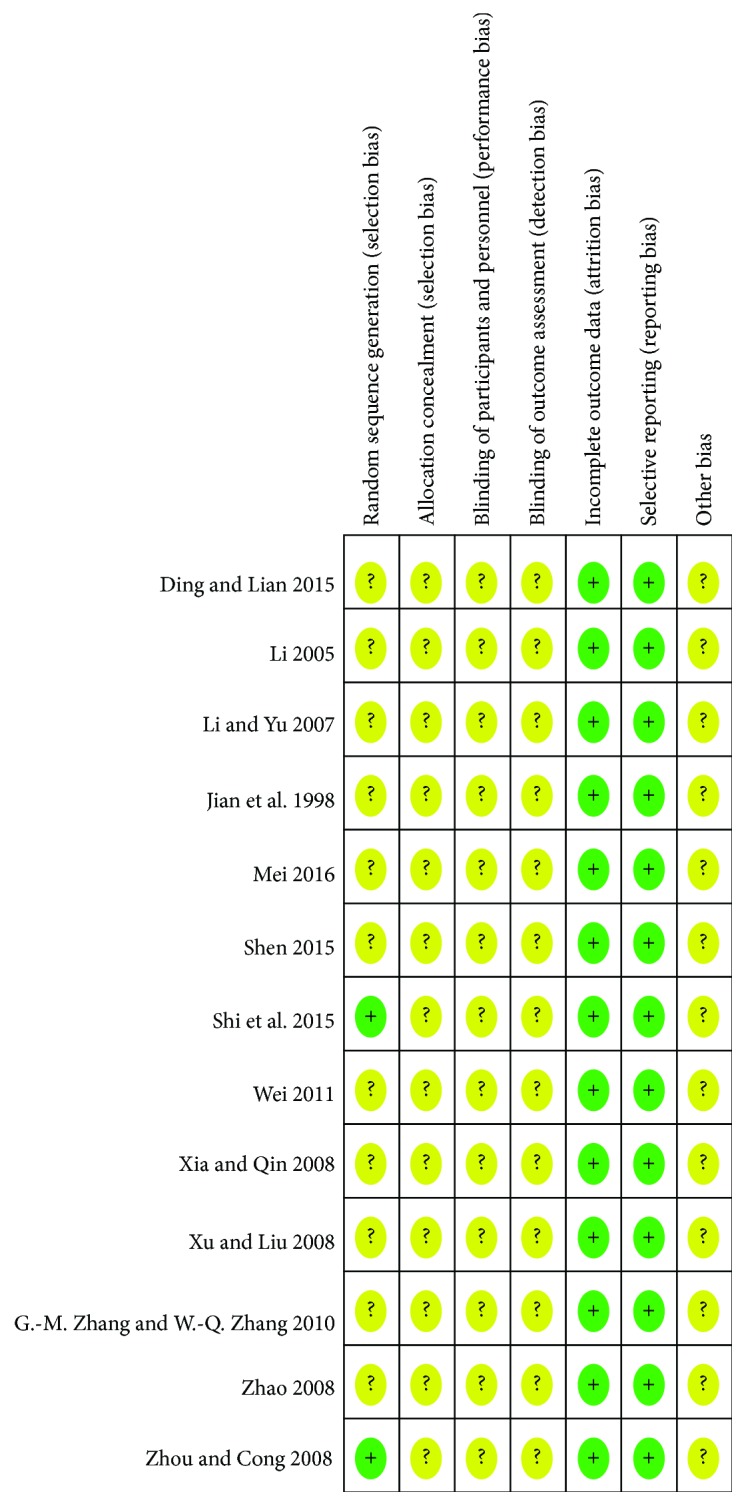
Risk of bias summary. +: low risk; −: high risk; ?: unclear risk.

In Figure 2, the methodology quality of the included trials was assessed as low. Although all of the trials were randomized, 3 reported the method of generating a random sequence (random number table) [21–23]. None of the trials reported any concealed allocation or blinding of patients and study participants. Of the 13 trials, 2 provided the number and reasons of dropouts [22, 24]. All of the relevant trials adequately addressed incomplete outcome data and selective reporting, and 4 trials included follow-up that ranged from 6 months to 1 year [20–22, 24].

## 4. Outcome Measures

### 4.1. Total Effectiveness Rates

**Figure 3:**
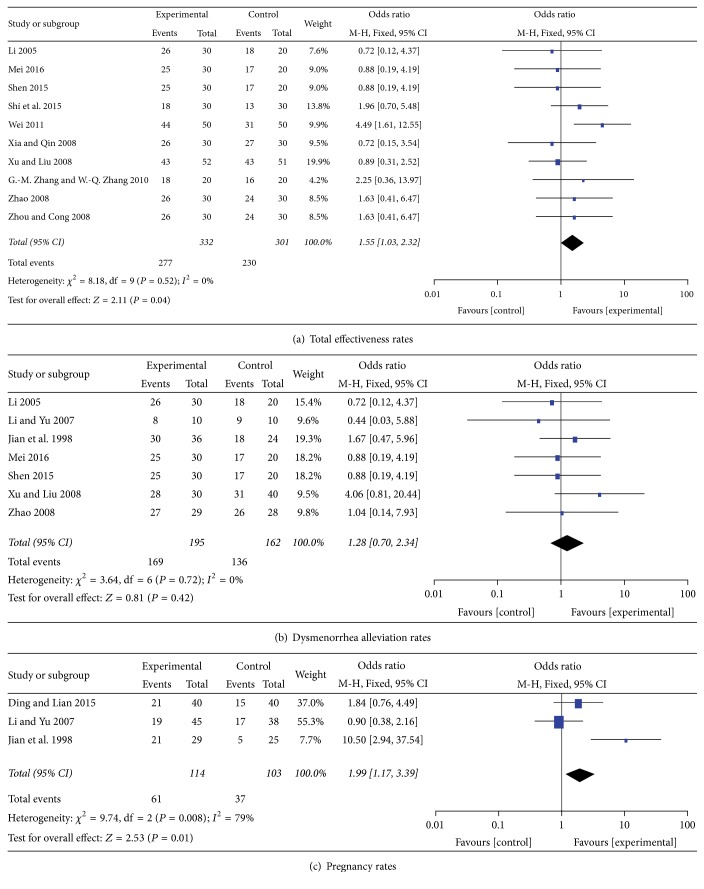
Meta-analysis of the total effectiveness rate, dysmenorrhea alleviation rate, and pregnancy rate of Bushen Huoxue prescription (BSHXP) versus Western medicine.

In 10 studies, 633 patients with endometriosis were included. The experimental and control groups received BSHXP and Western medicine, respectively. Heterogeneity tests involved a fixed-effects model to describe the data of the total effectiveness (*χ*^2^ = 8.18; *P* = 0.52; *I*^2^ = 0%). Our meta-analysis confirmed that the total effective rate of the BSHXP group was higher than that of the control group (RR, 1.55; 95% CI, 1.03–2.32; *P* = 0.04, Figure 3(a)).

### 4.2. Dysmenorrhea Alleviation Rates

In 7 studies, the effects of BSHXP and Western medicine on dysmenorrhea were compared and evaluated. Of the 314 patients with endometriosis included in these studies, 167 were included in the BSHXP group and 147 were assigned in the Western medicine group. The pooled results from these trials did not significantly differ in terms of the dysmenorrhea alleviation rates between the two groups (RR, 1.28; 95% CI, 0.70–2.34; *P* = 0.42, Figure 3(b)).

### 4.3. Pregnancy Rates

In 3 trials, pregnancy rates were reported. A total of 114 patients were included in the BSHXP group and 103 patients were assigned to the control group. Our meta-analysis showed that the pregnancy rates of the BSHXP group were higher than those of the Western medicine group (RR, 1.99; 95% CI, 1.17–3.39; *P* = 0.01, Figure 3(c)).

### 4.4. Change in Size of Endometriotic Cysts

In 6 studies, changes in the size of ectopic cysts were described. Three studies [24, 27, 29] compared the maximum cross-sectional areas of the cysts; two studies [20, 30] compared the maximum diameter line; one study [32] compared the reduction rates. Data could not be analyzed statistically because of inconsistent measurement standards. Thus, we only described the results of the original study.

In 2 studies [24, 29], the sizes of the cyst before and after treatment between BSHXP- and Western medicine-treated groups did not significantly differ (*P* > 0.05). Also the sizes of the cysts between the two groups did not also significantly vary after treatment (*P* > 0.05). In 4 studies [20, 27, 30, 32], the size of the cysts significantly differed (*P* < 0.05) before and after treatment between BSHXP- and Western medicine-treated groups. Nevertheless, the sizes of the cysts in these two groups did not significantly differ after treatment (*P* > 0.05).

Thus, our findings failed to support that TCM was more effective than Western medicine in reducing the size of endometriotic cysts.

### 4.5. Adverse Events

Six trials reported adverse events (6/14, 42.86%) [20, 21, 24, 29, 30, 32], whereas the other seven trials mentioned no significant adverse reactions (8/14, 57.14%) [21, 23, 25–28, 31]. In six trials, no evident adverse events occurred in the BSHXP group. In three trials, seven patients in the Western medicine group suffered from liver dysfunction with increased alanine transaminase (ALT) levels (10/102, 9.80%) [24, 29, 30]. In two trials [20, 30], 14 patients experienced hot flashes (14/60, 23.33%). In one trial [30], six patients had amenorrhea (6/30, 20.00%). In one trial [30], seven patients manifested acne (11/62, 17.74%). In one trial [21], two patients exhibited mood changes (2/30, 6.67%); three patients suffered from vaginal dryness (3/30, 10.00%), and three patients had decreased libido (3/30, 10.00%).

### 4.6. Publication Bias

**Figure 4:**
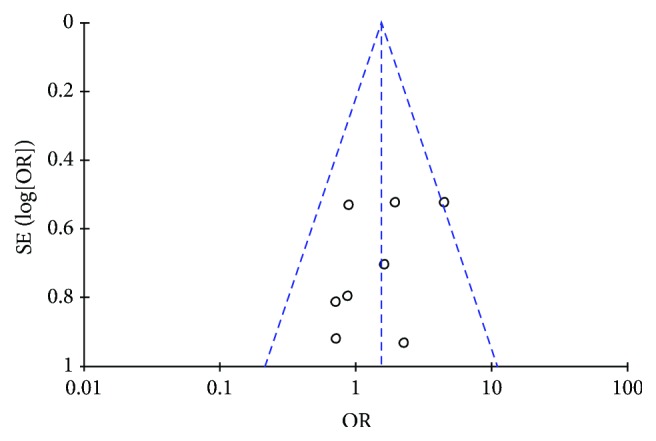
Funnel plot of the comparison of BSHXP versus Western medicine for the outcome of the total effectiveness rate.

The funnel plot of the 10 trials that compared BSHXP with Western medicine in terms of their total effective rates was analyzed to detect possible publication bias.  Figure 4 shows an asymmetrical funnel plot that indicates publication bias in the 10 selected articles.

## 5. Discussion

### 5.1. Summary of Evidence

A total of 936 patients with endometriosis were collected from the 13 RCTs in the review. The baseline of each study was consistent, and no statistical heterogeneity was observed. Our meta-analysis obtained the following results: (1) BSHXP did not significantly differ from Western medicine in terms of improving endometriosis-related symptoms and syndromes. (2) Similar to Western medicine, BSHXP emerged as an effective alternative therapy to alleviate endometriosis-associated pain. (3) BSHXP could significantly enhance the clinical pregnancy rate, but this conclusion should be further verified in future studies. (4) Studies have yet to provide evidence supporting that BSHXP is more effective than Western medicine in terms of reducing the size of endometriotic cysts. (5) The adverse effects of BSHXP were less reported than those of Western medicine, although six trials described serious events. Considering the number of the analyzed RCTs and the high risk of bias, we experienced difficulty in obtaining our conclusions.

### 5.2. Promising Alternative Therapy for Endometriosis

Long-term therapy is required for patients diagnosed with endometriosis. Current medical treatments may elicit side effects, such as inhibiting ovulation and menstruation and reducing estradiol to postmenopausal levels, because a definitive treatment for endometriosis has yet to be established [33, 34]. However, the natural history of this disease remains poorly understood [34]. As a chronic condition, endometriosis threatens the health of patients and entails high cost of care [35, 36]. Medical treatment should completely cure endometriosis rather than provide temporary relief from its symptoms [37]. In terms of endometriosis-associated infertility, new therapies have been developed to control pain symptoms without inhibiting ovulation [38]. New drugs replacing hormonal medicine for endometriosis will be available in the future, and these therapies should provide a normal and safe option for infertility [39]. In this regard, Chinese herbal medicines as complementary medicines are of great interest. Traditional Chinese medicine has been used to treat endometriosis. Tonifying kidney and activating blood stasis therapy may be a promising approach to treat endometriosis.

Considering syndrome differentiation and treatment system, doctors believe that endometriosis is mostly caused by kidney deficiency and blood stasis. Tonifying kidney and activating blood stasis prescriptions are designed. According to TCM theory, kidneys store essence and dominate reproduction. Chinese nourishing kidney herbs can regulate the hypothalamus-pituitary-ovary axis in dual directions and improve the functions of ovaries [40]. Liuwei Dihuang decoction, a well-known kidney-tonifying recipe, can enhance the rates of high-quality oocytes and embryos by regulating the microenvironment for oocytes of patients with endometriosis [41]. Ectopic cysts induce periodical bleeding defined in Chinese medicine as abnormal blood flow causing blood stasis. Activating blood herbs can promote blood circulation and alleviate blood stasis. Its treatment mechanism may be related to the regulation of immune conditions and the promotion of cell apoptosis in ectopic endometrium in vivo [42–44]. However, valid evidence, including meta-analysis results, has yet to be obtained for further recommendation. To our knowledge, this meta-analysis is the first research written in English to examine the effectiveness of BSHXP for endometriosis treatment.

## 6. Limitations

The methodologic quality of this review was generally poor. Although all of the studies were randomized, only two trials involved sequence generation [21, 23]. No trials reported allocation concealment. If patients and implementers were aware of the interventions, this meta-analysis would directly result in performance and detection biases. Seven trials reported adverse events, and these adverse events were described briefly. Therefore, the conclusion about the safety of BSHXP could not be obtained definitely.

This meta-analysis was also limited by various evaluating criteria among different studies. For example, three trials compared the maximum cross-sectional areas in terms of the size of cysts, two trials compared the maximum diameter, and one trial indicated the reduction ratio. Thus, we were unable to analyze these data uniformly. We suggest that researchers should use internationally unified diagnostic and scoring criteria in the future.

The combination of laparoscopy and histological verification is considered as a standard diagnostic technique for endometriosis. Only three trials demonstrated that all of the patients were diagnosed with laparoscopy. In other trials, some included in this review used a clinical diagnostic standard and others employed laparoscopic diagnosis. However, we were unable to divide laparoscopic- and clinical-diagnosed cases into two subgroups for sensitivity analysis because counting all of the cases of laparoscopic diagnosis in one study is impossible.

## 7. Conclusion

Our study provided evidence that BSHXP is effective and safe for endometriosis, but this evidence is inconclusive because of the low methodological quality of the included RCTs. Our findings suggest that BSHXP is an alternative drug for endometriosis, but it should be further examined in future clinical research.

## Figures and Tables

**Table 1:** Evaluation criteria on the efficacy of TCM symptom and syndromes recommended by GCRNDTCM.

Classification	Detailed description
Cure	(A) Clinical symptoms, gynecological examination, and B-ultrasound pelvic mass disappeared (B) No recurrence was found within 6 months
Markedly	Clinical symptoms disappeared or significantly reduced, with gynecological examination and B-ultrasound pelvic mass reduction of 2/3 or more
Effective	Clinical symptoms, gynecological examination, and B-ultrasound examination pelvic mass reduction 1/3 or more
Invalid	(A) Clinical symptoms, gynecological examination, and B-ultrasound pelvic mass had no significant change (B) Recurrence of progressive abdominal pain, and B-ultrasound reemergence of pelvic mass or mass has been reduced to grow up again

TCM, traditional Chinese medicine; GCRNDTCM, Guidelines of Clinical Research of New Drugs of Traditional Chinese Medicine.

**Table 2:** Basic characteristics of the included studies.

References	Sample size (T/C)	Age (T/C)	Baseline difference	Treatment	Control	Treatment duration	Adverse effects report	Main outcomes
Ding and Lian 2015 [20]	40/40	28.1 ± 2.1/28.6 ± 3.4	NSD	BSHXP (1 dose/day)	Mifepristone (12.5 mg/d)	6 months	Yes	Pregnancy rate, the serum level of CA125
Zhou and Cong 2008 [21]	30/30	38.80 ± 7.39/37.60 ± 7.3	NSD	BSHXP (1 dose/day)	Diphereline (3.75 mg once monthly)	6 months	Yes	Total effect rate, change in size of endometriotic cyst
Wei 2011 [22]	50/50	33.80 ± 8.58/33.80 ± 8.58	NSD	BSHXP (1 dose/day)	Gestrinone (2.5 mg/times, 2 times per week)	3 months	No	Total effect rate
Shi et al. 2015 [23]	30/30	29.4 ± 6.12/27.4 ± 5.56	NSD	BSHXP (1 dose/day)	Danazol (400 mg daily in 2 divided doses)	3 months	No	Total effect rate
Mei 2016 [24]	30/20	34.9 ± 5.5/36.0 ± 6.26	NSD	BSHXP (1 dose/day)	Mifepristone (12.5 mg/d)	3 months	Yes	Total effect rate, change in size of endometriotic cyst, dysmenorrhea alleviation rate, the serum level of CA125
Xu and Liu 2008 [25]	52/51	NR	NSD	BSHXP (1 dose/day)	Danazol (400 mg daily in 2 divided doses)	3 months	No	Total effect rate
Xia and Qin 2008 [26]	30/30	NR	NSD	BSHXP (1 dose/day)	Danazol (400 mg daily in 2 divided doses)	3 months	No	Total effect rate
Li 2005 [27]	30/20	35.14 ± 9.18/35.79 ± 9.32	NSD	BSHXP (1 dose/day)	Danazol (400 mg daily in 2 divided doses)	3 months	No	Total effect rate, dysmenorrhea alleviation rate, change in size of endometriotic cyst
G.-M. Zhang and W.-Q. Zhang 2010 [28]	20/20	31.5/32.3	NSD	TKAB-basic formula (1 dose/day)	Danazol (400 mg daily in 2 divided doses)	3 months	No	Total effect rate, the serum level of CA125
Shen 2015 [29]	30/20	36.13 ± 5.30/36.00 ± 6.26	NSD	TKAB-basic formula	Mifepristone (10 mg/d)	3 months	Yes	Total effect rate, dysmenorrhea alleviation rate, change in size of endometriotic cyst, the serum level of CA125
Zhao 2008 [30]	30/30	35.07 ± 6.70/35.97 ± 5.83	NSD	BSHXP (1 dose/day)	Danazol (400 mg daily in 2 divided doses)	3 month	Yes	Total effect rate, dysmenorrhea alleviation rate, change in size of endometriotic cyst
Jian et al. 1998 [31]	58/45	33.6/33.8	NSD	BSHXP (1 dose/day)	Danazol (600 mg daily in 3 divided doses)	3 month	No	pregnancy rate, dysmenorrhea alleviation rate
Li and Yu 2007 [32]	62/58	NR	NSD	BSHXP (1 dose/day)	Mifepristone (12.5 mg/d)	6 month	Yes	Total effect rate, dysmenorrhea alleviation rate, change in size of endometriotic cyst, pregnancy rate

T, treatment group; C, control group; NR, not reported; NSD, no significant difference; BSHXP, Bushen Huoxue prescription.

**Table 3:** Herbal medicines in the included studies.

References	Formula	Composition of formula
Ding and Lian 2015 [20]	BSHXP	Prepared Radix Rehmanniae 20 g, Dodder 20 g, *Angelica* 15 g, *Salvia* 15 g, Caulis Spatholobi 15 g, *Cyperus rotundus* 20 g, *Curcuma zedoaria *12 g, Chuan Cattle Cane 12 g, Poria Cocos 15 g, Cassia Twig 15 g, Rhizoma Corydalis 15 g, Trogopterus Dung 15 g, Red Peony Root 15 g
Zhou and Cong 2008 [21]	BSHXP	*Prunella vulgaris* 30 g, Raw Oyster 30 g, Calcined Corrugated Child 30 g, *Salvia chinensis* 18 g, *Codonopsis* 15 g, Sunburn Turtle 12 g, Antler Slices 9 g, *Eupolyphaga* 9 g, Leeches 6 g, Gun Ginger 6 g
Wei 2011 [22]	BSHXP	*Astragalus* 20 g, White Atractylodes Rhizome 10 g, Chinese Yam 15 g, *Dipsacus* 10 g, Dodder 10 g, Psoralen 10 g, *Angelica* 15 g, Red Peony 10 g, Safflower 10 g, Triangular 10 g, *Curcuma* 10 g, Pollen Typhae 10 g, Trogopterus Dung 10 g
Shi et al. 2015 [23]	BSHXP	Triad 9 g, *Curcuma* 9 g, Pilgrimage 12 g, *Eupolyphaga* 12 g, Cistanche 12 g, *Cuscuta* 12 g, Morinda 12 g, *Cynomorium* 12 g, Tortoise Plastron 18 g, Antler Slices 9 g, Aerial Parts of *Epimedium* 30 g, Hematoxylin 9 g, *Prunella vulgaris* 9 g
Mei 2016 [24]	BSHXP	Dodder 10 g, Placenta 10 g, *Angelica* 10 g, Szechuan Lovage Root 10 g, Citri Reticulatae Viride 6g, Red Peony 10 g, Corydalis Rhizome 10 g, *Euonymus alatus* 10 g
Xu and Liu 2008 [25]	BSHXP	Morinda 20 g, Aerial Parts of *Epimedium* 20 g, Chinese yam 15 g, *Cornus officinalis* 15 g, *Rehmannia* 15 g, Motherwort 15 g, Corydalis Rhizome 15 g, Combined Spicebush Root 15 g, Rhizoma Sparganii 10 g, *Curcuma zedoaria* 10 g
Xia and Qin 2008 [26]	BSHXP	Dodder 20 g, Eucommia 15 g, *Astragalus* 40 g, *Salvia* 20 g, Cinnamon 6 g, Red Peony 12 g, Trogopterus Dung15 g, Peach Kernel 9 g, *Cyperus rotundus* 12 g, Chicken Gizzard's Internal Lining 6 g, Poria 12 g, Cedar 12 g, licorice 6 g.
Li 2005 [27]	BSHXP	Dodder 30 g, *Astragalus* 30 g, *Salvia* 30 g, Eucommia 15 g, Cinnamon 6 g, Red Peony 12 g, Trogopterus Dung 15 g, Peach Kernel 9 g, Tree Peony Bark 12 g, Nut Grass Rhizome 12 g, Chicken Gizzard's Internal Lining 6 g, Poria 12 g, Liquorice Root 6 g
G.-M. Zhang and W.-Q. Zhang 2010 [28]	BSHXP	*Angelica* 10 g, Red Peony 19 g, Poria 10 g, Atractylodes 10 g, Chinese Yam 10 g, Trogopterus Dung 10 g, Eucommia 12 g, Dodder 12 g, Antler Cream 12 g, Corydalis Rhizome 15 g, Pangolin 1.5 g
